# Indoxyl sulfate and high-density lipoprotein cholesterol in early stages of chronic kidney disease

**DOI:** 10.1080/0886022X.2020.1845731

**Published:** 2020-11-16

**Authors:** Li Wang, Fangfang Xiang, Jun Ji, Xiaoqiang Ding, Bo Shen, Jing Chen, Yunqin Chen, Ning Xue, Lin Zhang, Xiaotian Jiang, Xuesen Cao

**Affiliations:** a Department of Nephrology, Zhongshan Hospital, Fudan University, Shanghai, China; b Shanghai Institute of Kidney and Dialysis, Shanghai, China; c Shanghai Key Laboratory of Kidney and Blood Purification, Shanghai, China; d Shanghai Institute of Cardiovascular Disease, Zhongshan Hospital, Fudan University, Shanghai, China

**Keywords:** Indoxyl sulfate, HDL-c, chronic kidney disease, cardiovascular disease

## Abstract

**Background:**

High IS level has been demonstrated to be associated with vascular calcification and lymphocyte functional disorders, which are both risk factors of CVD. Low HDL-c level is a risk factor of CVD in CKD patients. This study was designed to explore the potential relationship between IS and HDL-c levels in early stages of CKD population.

**Methods:**

Patients of CKD stage 1-3 were enrolled in this cross-sectional study. Correlations between HDL-c and IS levels were investigated among various clinicopathological variables through independent samples *t* test and multivariate logistic regression.

**Results:**

A total of 205 CKD patients (96 men) aged 43.27 ± 13.80 years old were included in this research. There were 96 patients (46 men) in CKD stage1 and 109 (50 men) in CKD stage 2 or stage 3. IS levels were significantly higher in CKD 2 + 3 group (1.50 ± 1.74 μg/ml *vs.* 0.94 ± 0.66 μg/ml, *p* = 0.007), while HDL-c levels were lower (1.19 ± 0.39 mmol/L *vs.* 1.33 ± 0.45 mmol/L, *p* = 0.017) compared to CKD 1 group. Among all the patients, a negative correlation was observed between IS and HDL-c levels (r = −0.244, *p* = 0.001). IS level was an independent risk factor for low HDL-c (<1.04 mmol/L) incidence even after controlling for potential confounders including concomitant disease, age, sex, blood pressure, BMI and laboratory biochemical test including eGFR (OR = 1.63, 95% CI: 1.11–2.39, *p* = 0.013). IS and HDL-c were both risk factors for predicting CKD stage 3.

**Conclusions:**

In early CKD stages, low HDL-c level is associated with increased IS levels, which may be an important contributor in the development of dyslipidemia in CKD patients.

## Background

Chronic kidney disease (CKD) is associated with higher mortality of cardiovascular disease (CVD) [1]. Indoxyl sulfate (IS), a protein-bound uremic toxin, is one of the organic anions that results from the metabolism of dietary tryptophan and after intestinal absorption is further converted to IS in the liver [2]. Microbiome and intestinal permeability changes induced by hypervolemia may lead to increased IS, inflammation and endothelial dysfunction [3,4]. IS is excreted *via* proximal tubular secretion in the kidney and it accumulates in the blood of patients with declined renal function. As one of the most extensively studied uremic toxins, IS may predict CKD progression [5]. Cao et al. [6] from our group reported that high serum IS level was associated with higher risk of first heart failure event in patients under hemodialysis. Previous studies [7,8] performed by Xiang and Chen at al. from our group have revealed the regulatory mechanism of IS on vascular calcification and lymphocyte functional disorders, which are both risk factors of CVD.

Epidemiological studies have shown that high-density lipoprotein cholesterol (HDL-c) level is independently and inversely correlated with CVD [9]. Reduced kidney function is associated with disruptions in the morphology and lipid metabolism [10–12]. Dyslipidemia in CKD is typically characterized by high triglyceride (TG) and low HDL-c levels [13]. A recently published study demonstrated that lower HDL-c is associated with atherosclerosis cardiovascular disease (ASCVD) in persons with CKD [14].

So there aroused the question that whether IS has an effect on HDL metabolism like on vascular calcification and lymphocyte functional disorder in CKD. This study was then designed to explore that if increased IS level was an independent risk factor for low HDL-c levels in early CKD stages, the results of which may provide a new intervention target on CKD dyslipidemia.

## Materials and methods

### Study population

From October 2012 to January 2014, stages of CKD1,CKD2 and CKD3 patients aged 18-70 years were enrolled from Department of Nephrology, Zhongshan Hospital, Fudan University.

Exclusion criteria included: (1) Dialysis therapy; (2) Obesity (BMI ≥ 30kg/m^2^); (3) Recent 3 months usage of drugs known to influence lipid metabolism; (4) Recent 3 months usage of drugs that scavenging toxins through the intestines, such as Coated Aldehyde Oxystarch; (5) Recently 3 months usage of glucocorticoid or immunosuppressants; (6)History of New York Heart Association class III/IV heart failure; (7) Acute infection; (8) Liver cirrhosis; (9) Severely elevated serum alanine aminotransferase(ALT) or aspartate aminotransferase (AST) levels (1.5 times higher than normal upper limit); (10) Malignant tumor; (11) Human immunodeficiency virus infection.

All patients provided written informed consent for participation in accordance with the Declaration of Helsinki. The study was approved by the hospital ethical review board (Zhongshan Hospital, Fudan University, Shanghai, China).

### Anthropometric measurements, blood sampling and clinical data collection

All patients were examined and blood sampling was performed in the morning after an overnight fast of 10–12 h. The date of birth, underlying kidney disease, past medical history were recorded. Height and weight (light clothes and without shoes), and resting blood pressure were determined by an experienced physician. Body mass index (BMI) was calculated as the weight in kilograms divided by the height in meters squared.

24h urine sample was collected for urine protein quantification under aseptic precautions from the day before interview.

### Biochemical measurements

Serum albumin, prealbumin, hemoglobin, blood urea nitrogen (BUN), serum creatinine (SCr), uric acid (UA), glycated hemoglobin (HbA1c), TG, total cholesterol (TC), HDL-c, low-density lipoprotein cholesterol (LDL-c) and high-sensitivity C-reactive protein (hsCRP) were measured using standard methods in the clinical laboratory.

Estimated glomerular filtration rate (eGFR) was calculated by the Chronic Kidney Disease Epidemiology Collaboration (CKD-EPI) equation.

Plasma IS concentration was detected using modified high-performance liquid chromatography (HPLC) tandem mass spectrometry method as described in our previous article [5].

### Statistical analysis

All variables were expressed as means ± SDs, or medians (interquartile ranges).

Comparisons between the 2 groups (CKD1 *vs.* CKD2 + 3) were assessed by independent samples *t* tests and *X*^2^-test (for categorical variables). Pearson/Spearman analysis was used to examine the correlation between IS and lipids levels and other biochemical variables.

Values of IS quartiles were defined as follows: (1) Q1: <0.54 μg/ml; (2) Q2: 0.54 μg/ml–0.88 μg/ml; (3) Q3: 0.88 μg/ml–1.59 μg/ml; (4) Q4: ≥1.59 μg/ml. Difference of HDL-c levels in the four IS quartile groups was tested by One-way ANOVA.

Odds ratios of low HDL-c (HDL-*c* < 1.04 mmol/L) occurrence with increased IS level were explored through multivariate longitudinal logistic regression model, in which IS values were all Ln transferred.

Factors predicting CKD stage 3 were also explored through multivariate longitudinal logistic regression model.

A two-tailed *p* < 0.05 was considered statistically significant. For all statistical analyses, SPSS Statistics 22.0 (IBM, Armonk, NY, USA) was used.

## Results

### Characteristics of study population

According to eGFR levels, 205 patients were divided into 2 groups: (1) CKD1 (eGFR ≥ 90 mL/min/1.73m^2^, *n* = 96); (2) CKD2 + 3 (30 mL/min/1.73m^2^≤eGFR < 89 mL/min/1.73m2, *n* = 109). Comparisons of clinical and biochemical characteristics between the 2 groups were shown in Table 1.

**Table 1.** Patients characteristics.

	Overall (*n* = 205)	CKD1 (*n* = 96)	CKD2 + 3 (*n* = 109)	*p* value
Demographics				
Age, y	43.27 ± 13.80	41.46 ± 13.84	44.87 ± 13.66	0.077
Sex (men/women)	96/109	46/50	50/59	0.781
Smoking History (%)	27 (13.2%)	15 (15.6%)	12 (11.0%)	0.409
Anthropometric Measurements				
Systolic BP, mmHg	131.48 ± 17.36	128.92 ± 13.87	133.73 ± 19.73	0.108
Diastolic BP, mmHg	82.87 ± 11.48	81.48 ± 10.19	84.09 ± 12.43	0.072
BMI, kg/m^2^	24.24 ± 3.69	24.23 ± 3.41	24.26 ± 3.93	0.948
Underlying Kidney Disease (*n*, %)				
Glomerular disease	164 (80.0%)	84 (87.5%)	80 (73.4%)	
Diabetic nephropathy	16 (7.8%)	3 (3.1%)	13 (11.9%)	
Hypertensive nephropathy	7 (3.4%)	2 (2.1%)	5 (4.6%)	
Polycystic kidney disease	1 (0.5%)	1 (1.0%)	0	
Others	1 (0.5%)	0	1 (0.9%)	
Unknown	16 (7.8%)	6 (6.3%)	10 (9.2%)	
Comorbidity (n, %)				
Hypertension	97 (47.3%)	33 (34.4%)	64 (58.7%)	0.001
Diabetes	29 (14.1%)	9 (9.4%)	20 (18.3%)	0.073
Gout	6 (2.9%)	2 (2.1%)	4 (3.7%)	0.687
CVD	7 (3.4%)	1 (1.0%)	6 (5.5%)	0.124
Laboratory Tests				
Hemoglobin, g/L	131.00 ± 19.17	136.45 ± 14.77	126.16 ± 21.29	<0.001
HbA1c, %	5.67 ± 0.94	5.54 ± 0.87	5.79 ± 0.99	0.047
Albumin, g/L	33.72 ± 8.52	33.84 ± 8.68	33.61 ± 8.43	0.842
pre-Albumin, g/L	0.29 ± 0.07	0.29 ± 0.06	0.29 ± 0.07	0.613
BUN, mmol/L	5.96 ± 2.76	4.60 ± 1.28	7.17 ± 3.13	<0.001
Scr, μmol/L	80.50 (64.0-108.8)	64.00 (55.5-76.5)	107.00 (85.3-130.3)	<0.001
UA, μmol/L	360.64 ± 99.00	328.04 ± 86.56	389.62 ± 100.69	<0.001
eGFR, ml/min/1.73m^2^	84.59 ± 26.20	107.88 ± 11.32	64.30 ± 16.92	<0.001
Urine protein, g/d	1.38 (0.74-2.81)	1.23 (0.69-2.67)	1.71 (0.95-3.16)	0.085
IS, μg/ml	1.24 ± 1.37	0.94 ± 0.66	1.50 ± 1.74	0.007
TC, mmol/L	5.56 ± 2.00	5.62 ± 2.04	5.52 ± 1.97	0.722
TG, mmol/L	1.77 (1.25-2.67)	1.72 (1.19-2.53)	1.91 (1.30-2.78)	0.533
HDL-c, mmol/L	1.25 ± 0.42	1.33 ± 0.45	1.19 ± 0.39	0.017
LDL-c, mmol/L	3.36 ± 1.70	3.33 ± 1.71	3.37 ± 1.69	0.871
hsCRP, mg/L	1.20 (0.43-2.38)	1.00 (0.48-2.20)	1.40 (0.40-2.63)	0.168

Continuous data expressed as mean ± standard deviation or median [interquartile range]; Categorical data expressed as count (percentage).

*p* value: CKD2 + 3 group vs. CKD1 group.

CKD: chronic kidney disease; BP: blood pressure; BMI: body mass index; HbA1c: glycated hemoglobin; BUN: blood urea nitrogen; Scr: serum creatinine; UA: uric acid; eGFR: estimated glomerular filtration rate; IS: indoxyl sulfate; TC: total cholesterol; TG: triglycerides; HDL-c: high-density lipoprotein cholesterol; LDL-c: low-density lipoprotein cholesterol; hsCRP: high- sensitivity C-reactive protein.

Age and sex were equally matched (both with *p* > 0.05). There was no significant difference of blood pressure and BMI between the study groups. Prevalence of hypertension was higher in group CKD2 + 3 (58.7 vs. 34.4%, *p* = 0.001). Compared to CKD1 group, CKD2 + 3 group presented higher levels of HbA1c (5.79 ± 0.99 vs. 5.54 ± 0.87%), UA (389.62 ± 100.69 vs. 328.04 ± 86.56 μmol/L), IS (1.50 ± 1.74 vs. 0.94 ± 0.66 μg/ml) and lower levels of hemoglobin (126.16 ± 21.29 vs. 136.45 ± 14.77 g/L) and HDL-c (1.19 ± 0.39 vs. 1.33 ± 0.45 mmol/L) (all with *p* < 0.05).

### The association between is and HDL-c as well as other variables

As shown in Table 2, serum IS levels were positively correlated with systolic BP, diastolic BP, hypertension history, CVD history, levels of albumin, BUN, Scr and hsCRP and negatively correlated with eGFR, hemoglobin, HDL-c and urine protein levels (all with *p* < 0.05).

**Table 2.** Correlation of high IS levels with other variables.

Variables	r	*p* value
eGFR	−0.245	<0.001
Systolic BP	0.221	0.001
Diastolic BP	0.143	0.040
Hypertension History	0.216	0.002
CVD History	0.230	0.001
Hemoglobin	−0.157	0.025
Albumin	0.280	<0.001
BUN	0.242	<0.001
Scr	0.222	0.001
HDL-c	−0.244	0.001
hsCRP	0.168	0.019
Urine protein	−0.254	<0.001

IS: indoxyl sulfate; HDL-c: high-density lipoprotein cholesterol; CVD: cardiovascular disease; eGFR: estimated glomerular filtration rate; BP: blood pressure; BUN: blood urea nitrogen; Scr: serum creatinine; hsCRP: high- sensitivity C-reactive protein.

**Figure 1.**
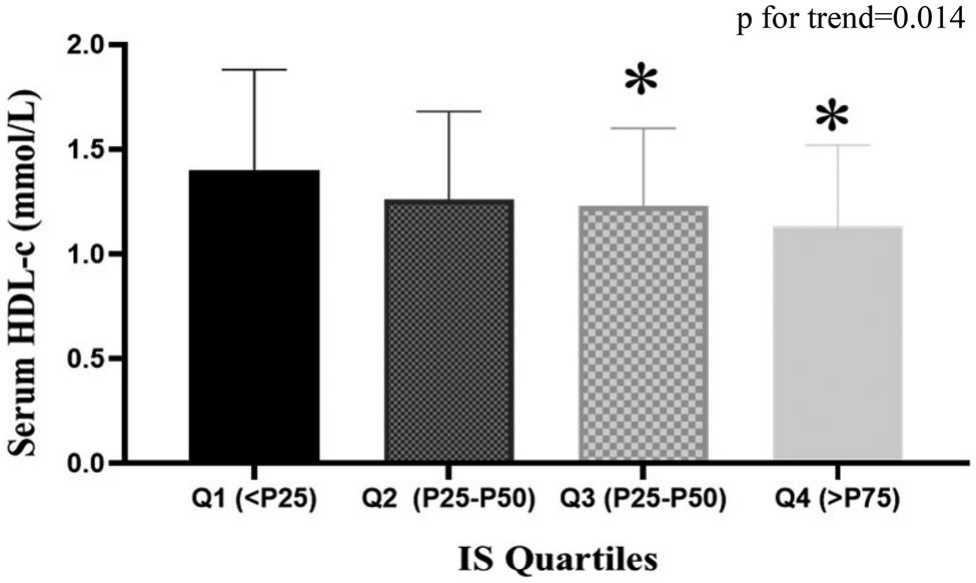
Trend of HDL-c levels in the four IS quartiles.* vs. quartle1, *p* < 0.05.

The subjects were then divided into four groups according to the quartile values of IS (Q1: IS < P25, Q2: P25 ≤ IS < P50, Q3:P50 ≤ IS < P75, Q4:IS ≥ P75). Figure 1 showed that as IS levels increased, HDL-c levels decreased from group to group. Serum HDL-c level in each group was 1.40 ± 0.48, 1.26 ± 0.42, 1.23 ± 0.37, 1.13 ± 0.39 mmol/L respectively (p for trend = 0.014).

### Impact of is levels on risk of low HDL-c levels incidence

Table 3 lists the risk of low HDL-c levels (defined as HDL-*c* < 1.04 mmol/L) incidence as IS levels increased [OR = 1.56, 95%CI (1.07–2.27), *p* = 0.018] (Model 1). After adjustment for medical history of hypertension, diabetes, gout and CVD, age, sex, Systolic BP, Diastolic BP and BMI (Model 2), IS showed an odds ratio of 1.55 [95%CI (1.05–2.30), *p* = 0.028]. After further adjustment for hemoglobin, HbA1c, albumin, hsCRP, eGFR, BUN, Scr, UA and 24 h urine protein(Model 4), IS still showed a significant OR of 1.57[95%CI (1.02–2.41), *p* = 0.039].

**Table 3.** Logistic regression of low HDL-c incidence with IS levels increment.

	OR (95%CI)	*p* value
Model 1	1.56 (1.07–2.27)	0.018
Model 2	1.55 (1.05–2.30)	0.028
Model 3	1.71 (1.13–2.59)	0.010
Model 4	1.57 (1.02–2.41)	0.039

Model 1. Crude;.

Model 2. Adjusted for hypertension history, diabetes history, gout history, smoking history and CVD history, age, sex, Systolic BP, Diastolic BP and BMI;.

Model 3. Model 2 further adjusted for hemoglobin, HbA1c, albumin and hsCRP;.

Modle 4. Model 3 further adjusted for eGFR, BUN, Scr, UA and 24 h urine protein.

### Risk factors predicting for CKD stage 3

Table 4 shows that HDL-c [OR:0.15, 95%CI (0.04–0.57)], IS[OR:1.67,95%CI (1.06–2.63)], systolic BP [OR:1.04 (1.01–1.06)], hemoglobin [OR:0.98 (0.95–1.00)] and urine protein [OR:1.23 (1.08-1.40)] levels were risk factors predicting for CKD stage 3.

**Table 4.** Risk factors predicting for CKD stage 3.

variables	OR(95%CI)	*p* value
HDL-c	0.15 (0.04–0.57)	0.006
IS	1.67 (1.06–2.63)	0.027
Systolic BP	1.04 (1.01–1.06)	0.003
Hemoglobin	0.98 (0.95–1.00)	0.004
Urine protein	1.23 (1.08–1.40)	0.002

Adjusted for medical history of primary hypertension, diabetes, gout and CVD, sex, age,

BMI, diastolic BP, HbA1c, albumin, pre-albumin, TG, TC, LDL-c and hsCRP.

CKD: chronic kidney disease; HDL-c: high-density lipoprotein cholesterol; IS: indoxyl sulfate; BP: blood pressure.

## Discussion

CKD is correlated with an increased risk of CVD as disease progresses [13,14]. Patients under dialysis have an extremely high risk of cardiovascular events [1]. Actually, relationship between CKD and CVD is present even under minor renal injury. However, most studies have focused on CVD risks mostly when eGFR is lower than 60 mL/min/1.73 m^2^ [1,15,16], In all relevant studies published to date, CVD is the predominant cause of increased mortality, accounting for over 50% of all deaths[1,15,17,18].

In general population, every 1 mmol/L (40 mg/dl) elevation in LDL-c level may result in an increased risk of CVD by 40% [19,20]. While in CKD patients, levels of residual renal function and proteinuria as well as comorbidities (especially type 2 diabetes) and treatment can all affect lipid metabolism [21,22]. However, the relationship between lipid profiles and CVD risks in CKD patients remains uncertain. In dialysis patients, serum LDL-c level has a negative association with all-cause mortality [23,24], the phenomenon of which is called ‘reverse epidemiology’. Low serum HDL-c levels are common among patients with CKD and ESRD [25–27]. Archna Bajaj et al. [14] recently reported that HDL-c was associated with increased risk for ASCVD beyond LDL-c among individuals with CKD.

Atheroprotective functions of HDL include anti-thrombotic activities [28] and endothelium regenerative capabilities [29,30], anti-inflammatory and anti-oxidative properties [31,32]. Innumerable studies have revealed that HDL metabolism is complex and involving multiple pathways. The process of HDL biogenesis is mediated primarily by the liver. ApoA-1 is the major lipoprotein on HDL which stimulates cholesterol efflux through ATP-binding cassette (ABC) transporters. The movement of cholesterol from peripheral tissues to the liver for clearance is termed reverse cholesterol transport (RCT), a pathway that represents a key atheroprotective function of HDL. Defective maturation of HDL particles, impaired Apo-A1-mediated cholesterol efflux, and limited RCT have been revealed in CKD patients [33].

As one of the most extensively studied protein-bound uremic toxins, IS may be associated with CVD and mortality in CKD patients. The process of IS biogenesis is mediated mainly in the liver [2,34–37]. More and more attention has been focused on the relationship between IS levels and CVD incidence among CKD population in recent years [38–42]. Taki et al. [43] found that high serum IS level was significantly correlated with incidence of atherosclerosis. Cao et al. [6] from our group reported that high serum IS was associated with higher risk of first failure event in patients on hemodialysis.

It is known that progressive decline of renal function is associated with increased IS and decreased HDL-c levels. This study firstly found an association between IS and HDL-c independent of renal function in early CKD stages. Besides the negative correlation, IS was an independent risk factor of low HDL-c incidence. Even after adjusting related conventional factors such as age, sex, BMI, history of diabetes, history of primary hypertension, history of coronary heart disease, blood pressure and so on, the OR value remained statistically significant as we expected. However, more basic researches are needed to confirm whether IS has a direct effect on any step of RCT, ApoA-1 mediated cholesterol efflux, HDL biogenesis and maturation, the results of which might bring new target on dyslipidemia therapy in CKD patients.

Smoking and obesity are known as related factors to dyslipidemia. In this study, percentage of smoking patients was small and statistically equal in the two study groups. No obvious correlation was found between smoking and IS or HDL-c levels. As for obesity, we did exclude obesity patients (BMI ≥ 30kg/m^2^). Though BMI in the two groups was matched, it was actually correlated with HDL-c (r = −0.232, *p* = 0.001). However, the association between IS and HDL-c remained meaningful after adjustment of BMI. Even after adjustment of age, sex, diabetes history, hypertension history, CVD history, HbA1c *et al*, the association was still meaningful. Therefore, we think that IS’s impact on HDL-c among CKD patients is independent on conventional risk factors.

There’s another important finding in this article that a negative correlation was found between proteinuria and IS (r = −0.254, *p* < 0.001). Definite mechanism has not been found through literature review. What has been already known is that glomerular proteinuria level decreases gradually as renal function declines with/without obviously reduced urine volume, so that there’s a positive correlation between eGFR and urine protein quantity. While IS level is negatively correlated with eGFR, so statistically we may consider that IS level may be negatively correlated with urine protein quantity. It’s not clear whether IS has a direct affect on glomerular pathological changes, which might inhibit protein excretion from kidney. More basic researches are needed to explore the mechanism of this finding.

There were still several limitations in this study. Firstly, the sample size was relatively small as a cross-sectional research. Secondly, the tested lipid contents (only TC, TG, HDL-c and LDL-c included) were not adequate to make omni-directional exploration of the relationship between lipid metabolism and IS in patients with CKD.

Our data revealed that low HDL-c level occurs in early stages of CKD, which might be resulted from increased IS level as the CKD stage worsens. This negative correlation exits between IS and HDL-c independent of GFR. Thus clinically, methods to reduce serum IS level (e.g., avoidance of hypervolemia induced microbiome and intestinal permeability changes, and use of uremic toxin adsorbent such as the oral charcoal adsorbent AST-120 [44,45]) might improve HDL-c metabolic disorder. Detailed mechanisms need to be further investigated.

## Data Availability

The datasets used and analyzed during the current study are available from the corresponding authors on reasonable request.
